# The Love of Man to God

**Published:** 1889-08-10

**Authors:** 


### Words of Consolation.

### THE LOVE OF MAN TO GOD.

(*For Reading to the Sick.*)

Next to contemplating the love of God to man it is good to dwell on the love of man to God. We have seen that the former is protective, indulgent, infinite, and we can never with our best efforts equal these manifestations of the Deity, but in our Lord's answer to the lawyer who tempted Him He tells man for all time what is required of him and what he is able to do: "Thou shalt love the Lord thy God with all thine heart, with all thy soul, and with all thy mind. This is the first and great commandment. And the second is like unto it, thou shalt love thy neighbour as thyself." Our path, then, is clear before us, and it is only our perverseness and want of humility which rouses our ill tempers and make us deem this dependence on and love to God an unnecessary thing. But to compare lesser things with greater, let us remember what happiness there is in life when loving hearts beat in sweet accord, when the husband and father rules with love and wisdom, and the wife, and children, and servants love, honour, and obey the master, whose chief care is for the well-being of all under his charge.

This is a type of the relationship there should be between God and man, between Christ and His Church. The infinite love, the protective care with which we are guarded and guided, should be met and reciprocated by us to the utmost of our power and intellect. The more we meditate on the glorious perfections of the Almighty, the more shall we appreciate and adore them; the longer we contemplate His divine love, the sooner shall we grow like him, and not until we know Him as He is shall we attain to that perfect bliss which is promised to them that love Him.

How much happiness is missed by the young who throw off all control as soon as possible, and squander their talents and time in pleasure and on those outside the home circle. It is well for them when sickness and sorrow come if they can creep back to the nest, and so learn to value what they had formerly despised.

In like manner do we try to get from under the restraining influences of religion. We like to have our fling, to feel we are free to owe obedience only to our own sweet wills; and so we go on flourishing for a time, and think our "will is so strong it cannot be removed." Then the loving Father steps in and stays our downward course. He takes away our health or our strength, which have not been dedicated to His service; or He removes the earthly ties which have held us away from Him, and at length we feel our weakness and our need. Happy for us is it if we kiss the chastening rod which comforts even while it punishes, and turning to Him with all our hearts find rest for our souls. Sometimes He hides His face from us for awhile, and we seem forsaken and "poor and naked and miserable," but it is only to draw us back by the cords of love until we fall in deep repentance and humble trust before the Divine footstool. Then what joy, what bliss, fills the soul when it is reconciled to its Maker! The heart burns and glows with love and praise to Him who is all in all. We believe in Him, we fear to offend Him, we love Him with all our hearts, with all our mind, with all our soul, and with all our strength; and we determine, with His help, to serve Him faithfully all the days of our life. Let us dedicate the remainder of our time in fervent zeal to His service, lest we draw on us the reproach cast on the Laodiceans for their lukewarmness, "I would that thou wert either hot or cold"; and so, having done our duty to God and our neighbours, as the Church Catechism quaintly puts it, we shall attain happiness in this world and bliss in the world to come.

---

Mr. H. F. Pease, M.P., made a capital speech at the hospital demonstration at North Ormesby. The meeting was held in the pretty grounds of the Cottage Hospital on July 28, and was largely attended. Mr. Pease said he agreed with the remarks of Lord Randolph Churchill, who said at a hospital meeting not long ago that the main features of the hospital institutions of this country were that they were voluntary, free, and independent; and these three qualities of hospital work were dear to the hearts of Englishmen, and especially to the North Riding workmen, who loved free and independent institutions which did not pauperise those who came within their walls.

